# Perioperative Arthroscopic Tourniquet Usage: Practices and Trends of the AANA Membership Are Varied

**DOI:** 10.1016/j.asmr.2025.101106

**Published:** 2025-02-20

**Authors:** Brian Day

**Affiliations:** Department of Orthopaedics, University of British Columbia, Vancouver, Canada

## Abstract

**Purpose:**

To investigate current practices for tourniquet use, including tourniquet times, indications, and other perioperative applications, among Arthroscopy Association of North America (AANA) members and to determine whether recommendations can be made for safer tourniquet use.

**Methods:**

Participants in this study were AANA members who responded to our survey in the AANA DocMatter Community. The survey was developed based on discussion prompts in DocMatter. The survey consisted of 28 questions on 4 core themes, including demographic characteristics, general understanding of tourniquet safety, applied tourniquet use in arthroscopy, and tourniquet technology in perioperative applications. The survey was open for responses for 7 weeks from September to November 2023. Multiple-choice responses were counted, and short-form responses were reviewed and aggregated based on keywords. Qualitative analysis was used to understand the data.

**Results:**

A total of 59 AANA DocMatter Community Members participated in the survey. Eighty percent of respondents had been in practice for more than 10 years, whereas 20% had been in practice for between 3 and 10 years. Tourniquet usage in arthroscopy was varied, although some patterns were detected. For example, 91% of responders applied tourniquets for all limb procedures, but many inflated them only as needed, and some preferred not to use a tourniquet. When tourniquet times were minimized by selectively inflating cuffs, tourniquet times averaged between 15 and 20 minutes compared with 20 to 60 minutes for continuous inflation. However, 76% of participants reported encountering venous congestion when a tourniquet was applied but not inflated. Perioperative tourniquet uses were also varied. Almost half of respondents used tourniquets for blood flow restriction therapy in their care protocols. In a separate possible trend, a majority of respondents were interested in using tourniquet-related technology to help resolve postsurgical lymphedema.

**Conclusions:**

Despite large reported variation in the indications for tourniquet use and the control of key tourniquet parameters, use is evolving through a focus on optimizing tourniquet times through selective inflation for specific aspects of arthroscopic procedures.

**Clinical Relevance:**

Standardized approaches to the safe use of tourniquets and their evolving applications are important areas of investigation.

Clinical tourniquet use applications are evolving. Clinicians seek to reduce potential risks by reducing tourniquet times and pressures. Guidelines on usage and technology continue to develop, with the goal of improved patient safety and outcomes.

Devices that restrict or occlude blood flow by compression of the limb and its underlying vessels have been in use for thousands of years. They were initially used to prevent death from hemorrhage, and survival—rather than limb safety—was the prime consideration.^1^ Tourniquets were later designed and used for controlling blood flow to enable a clearer field of vision during surgery.^2^ Major improvements in tourniquet technology and safety have been introduced over the years. The modern pneumatic surgical tourniquet is controlled via a microprocessor, which accurately maintains and regulates a calibrated set pressure.^3^ It incorporates many self-monitoring alarms that enhance patient safety. This evolution has improved many of the safety concerns of uncontrolled pneumatic and non-pneumatic tourniquet devices previously in use. Further safety innovations have included the ability to determine a patient’s limb occlusion pressure, which is the minimum pressure required to stop blood flow distal to a cuff applied to a patient’s limb.^4^, ^5^, ^6^

The benefits of tourniquets in scheduled limb operations largely relate to improved efficiencies from better visualization. In cases of trauma, the tourniquet still serves its historical role in life-saving blood control measures. Prehospital applications include the use of surgical-grade tourniquets for blood flow restriction (BFR) therapy, as well as emergency and military tourniquet use in the field, in which rapid, 1-handed self-application to control hemorrhage is often needed.^7^, ^8^, ^9^ Tourniquets for use in BFR therapy restrict arterial blood flow while a patient performs low-intensity exercise. This elicits physiological adaptations leading to increased muscle size, strength, and endurance, and load-compromised patients waiting for or recovering from surgery can benefit from BFR.^10^, ^11^, ^12^

The literature on safety guidelines and the implementation of perioperative standards for tourniquet use have not kept pace with the increased applications and the major improvements in technology. Studies over the past 20 years have shown large variations in key parameters such as cuff size and shape, use of a limb protection sleeve, cuff snugness, and tourniquet pressures and times, as well as how these key parameters can be optimized to retain the benefits of a tourniquet while mitigating risks and improving patient outcomes.^13^, ^14^, ^15^, ^16^, ^17^, ^18^

The purposes of this study were to investigate current practices for tourniquet use, including tourniquet times, indications, and other perioperative applications, among Arthroscopy Association of North America (AANA) members and to determine whether recommendations can be made for safer tourniquet use. It is hypothesized that there might be substantial variability in the understanding of the factors impacting tourniquet safety and effectiveness and that respondents would widely vary in their average tourniquet times and the procedures for which they used tourniquets.

## Methods

All participants in this study were AANA members who responded to prompts for discussion and/or completed the final survey in the organization’s online DocMatter Community. An initial forum discussion was posted in July 2023 to the AANA-exclusive DocMatter Community platform by the author to prompt AANA members to casually discuss current practices regarding tourniquet use preoperatively, intraoperatively, and postoperatively. Initial questions in this forum prompt included (1) the percentage of cases in which tourniquets are used, (2) whether tourniquet practice has changed over the past 10 years, (3) whether tourniquet times have become shorter or longer, (4) whether AANA Community members are familiar with BFR tourniquets, and (5) whether there is a need for a summary of current information on tourniquet usage in arthroscopy. The discussion prompt was open for 2 weeks and gathered 82 responses from the community.

On the basis of the feedback and questions raised from the AANA DocMatter Community members regarding the initial forum discussion, a more in-depth survey was developed to address the comments and questions raised from the initial forum discussion. Unlike more formal structured surveys that are circulated to a specified group, this survey was posted to the AANA DocMatter Community Forum and recruitment was based on community engagement. The survey consisted of 28 questions posed in a combination of multiple-choice and short-answer formats. The DocMatter team posted the survey on the community forum available to AANA Community members, with the survey open for response for a period of 7 weeks from September to November 2023.

The survey questions were organized and ordered into 4 core themes: demographic characteristics of AANA members, general understanding of tourniquet safety criteria, applied tourniquet use in arthroscopy, and tourniquet technology in perioperative applications. Multiple-choice responses were counted, and short-form responses were reviewed and aggregated based on keywords. Given the less controlled nature of the survey circulation, limited sample size, and format of short-form questions, qualitative statistics were used for these results.

## Results

A total of 59 AANA members responded to the final in-depth survey. Eighty percent of respondents were in practice for more than 10 years, whereas 20% were in practice for between 3 and 10 years. Detailed results are shown in Table 1, Table 2, Table 3. Given the short-form response nature of some of the survey questions, some questions and responses did not yield sufficient information to be able to aggregate results for all questions.

**Table 1:** Demographic Characteristics and General Understanding of Tourniquet Safety Criteria

Question	No. of Responses Received	Results
Years of arthroscopic practice	59	>10 yr: 80% 3-10 yr: 20%
Preference of tourniquet use	59	Use: 42% Apply cuff, inflate as needed: 32% Not use: 26%
Understanding that reducing tourniquet times reduces risks	59	Yes: 92% No: 5% Unsure: 3%
Understanding that reducing tourniquet pressures reduces risks	59	Yes: 85% No: 10% Unsure: 5%

**Table 2:** Tourniquet Use in Arthroscopy

Question	No. of Responses Received	Results
Reasons to inflate a tourniquet	58	Improved visualization: 100% Better blood flow control: 43% Faster operating time: 53% Use in training: 21%
Reasons to not a inflate tourniquet	52	Other techniques to achieve visualization: 60% Insignificant difference in blood loss: 38% Improved patient outcomes: 38% Shorter recovery times: 29%
Procedures for which a tourniquet might be used	57	Ligament reconstruction, graft harvesting, and meniscus repair: 44% Limb extremity scope procedures: 33% None: 23%
Specific aspect of a procedure for which the tourniquet is inflated	48	ACL graft harvest: 38% Ligament reconstruction: 17% Synovectomy: 6% Other Limit pump pressure: 2% Bone violation: 2% steotomy: 2% General visualization: 14% Not applicable: 19%
Techniques to achieve hemostatic control	55	Primary techniques Epinephrine in irrigation: 69% Tourniquet: 84% Tranexamic acid: 56% Secondary techniques Trendelenburg: 5% Circumferential thigh holder: 5% Cautery: 4% Controlled outflow hypotension: 2% Radiothermic probe: 2% Hydrostatic pressure in irrigation fluid: 2%
Hemostatic control techniques to avoid	17	Circumferential thigh holder: 35% Epinephrine in irrigation fluid: 35% Tranexamic acid: 18% Tourniquet: 12%
Experience complications related to tourniquet use	52	No: 23% Yes: 77% Percent of these cases resulting in a concern <5%: 65% 20%-30%: 15% Not described: 20%
Venous congestion when a tourniquet is uninflated	50	Yes: 76% No: 20% Rarely: 4%

ACL, anterior cruciate ligament.

**Table 3:** Perioperative Tourniquet-Related Uses

Question	No. of Responses Received	Results
Use of blood flow restriction therapy in prehabilitation and rehabilitation care	46	Yes: 48%
		No: 52%
		Reason
		No benefit/risk concerns: 65%
		Lack of access/leave to physiotherapist: 35%
Occurrence of extravasation	40	Yes/conditional: 27%
		Rarely: 28%
		No: 15%
Occurrence and methods to decrease likelihood of postoperative swelling	43	Occurrence
		Frequent: 50%
		Infrequent: 50%
		Methods
		Ice: 16%
		Compression: 47%
		Elevation: 44%
Interest in tourniquet technology to resolve postsurgical lymphedema	42	Interested: 60%
		Low incidence—not interested: 40%

### Optimization of Tourniquet Time and Pressure

Ninety-one percent of surgeons applied a tourniquet cuff as part of routine procedure preparation, regardless of whether they intended to inflate the tourniquet. When the tourniquet was selectively inflated for specific aspects of a procedure, 41% of respondents indicated tourniquet times of less than 20 minutes; 37%, between 21 and 30 minutes; 11%, between 31 and 60 minutes; and 11%, more than 60 minutes. Separately, 12 surgeons did not report an average time, instead indicating the time was dependent on the procedure and required visualization. In comparison, when the tourniquet was used for the duration of the procedure, 35% of respondents indicated tourniquet times of less than 20 minutes; 15%, between 21 and 30 minutes; 41%, between 31 and 60 minutes; and 9%, more than 60 minutes.

Forty-two percent of surgeons indicated they do not apply techniques to adapt tourniquet pressure for individual patients. Among the 58% of surgeons who do apply such techniques, 62% rely on blood pressure and 48% rely on the limb circumference size to determine their desired tourniquet pressure.

## Discussion

The most important finding of this study is the substantial variation in the use of tourniquets among the participants, along with an interest in the safe use of tourniquets. Reducing tourniquet pressure and inflation time can reduce the risk of tourniquet-related injury, the details of which have been described in research elsewhere.^5^^,^^13^^,^^14^ Although the results indicate that there is wide variation in average tourniquet inflation times and that time is largely dependent on the surgical procedure, surgeons are moving toward selectively inflating the tourniquet to optimize tourniquet times.

To minimize tourniquet time, many surgeons apply a tourniquet cuff but inflate it only if bleeding becomes a problem. The respondents cited improved visualization and blood control, faster operating time, and an aid in teaching. Most inflated tourniquets for graft harvesting, synovectomy, bony procedures, and specific issues with bleeding or visualization. When tourniquets were inflated only as needed, the most commonly reported inflation times were 15 to 20 minutes. In contrast, when tourniquets were inflated for the duration of the procedure, times ranged between 20 and 60 minutes. A significant number of AANA members are reducing tourniquet time by identifying specific aspects of a procedure and inflating the cuff only as needed, believing this reduces risks while maintaining benefits.

Over 75% of respondents reported venous congestion when the cuff is applied but not inflated. Five surgeons believed that venous congestion also occurs when the tourniquet cuff is too snugly applied. This may be less impactful when a tourniquet is inflated for the complete procedure. Given the practice of decreasing tourniquet time by inflating the cuff only as required, the significance and safety implications of proper application snugness may increase.

Cuff snugness is important because both too tight and too loose applications can negatively impact safety and effectiveness of use.^19^ If the cuff is loose, it can shift on the limb and require higher pressures to occlude blood flow. Tight cuffs can cause venous congestion and related hazards and can increase the risk of port occlusion error in the cuff. Snugness levels can be inconsistent because of variables such as user training, cuff size, limb characteristics (muscle tone and limb shape), and cuff-securing method (tie ribbons vs application handle). The Association of Surgical Technologists Guidelines for Best Practices for Safe Use of Pneumatic Tourniquets describes the “finger test” method to verify cuff snugness.^20^ This method unfortunately does not overcome some of the inconsistencies that the fit variables introduce. If a cylindrical cuff is applied to a taper-shaped limb, a different number of fingers will be able to fit under the proximal cuff edge compared with the distal cuff edge. Furthermore, for very tapered limbs, a large distal gap may be unavoidable, resulting in a gap that can fit more than the recommended number of fingers. Finally, the instruction for “fingers to easily slide under the cuff edge” can be subjective; owing to the compressibility of soft tissue, any number of fingers may always be easily slid under a cuff edge at varying degrees of ease. Therefore, in addition to the need to develop improved guidelines for awareness and training of proper cuff application, there may also be a possible opportunity for better cuff technology to match the evolving advances in tourniquet use.

Despite the vast majority of respondents understanding that reduced tourniquet pressures may lower risks, over 40% did not use personalized pressures. Those who attempted to personalize tourniquet pressure used blood pressure and limb size as 2 variables to consider. Other studies have shown that blood pressure and limb size alone are not significantly correlated to the limb occlusion pressure, which is the minimum pressure required for a specific patient with a specific cuff measured at a specific time to occlude distal blood flow.^5^^,^^21^^,^^22^

The 3 most commonly cited methods to achieve hemostatic control were use of a tourniquet (83%), epinephrine in the irrigation fluid (69%), and tranexamic acid (56%). However, when surgeons were asked about which techniques they would avoid, responses also included use of a circumferential thigh holder because of venous tourniquet effect, epinephrine in fluid due to bleeding or impacts on articular cartilage, and tranexamic acid for patients with cardiac risks. Each of these methods has its own risk-benefit profile that varies with the surgical procedure, the patient, and surgeon preference, and each method has key parameters that should be optimized to reduce the associated risks.

Although the survey was primarily focused on tourniquet use in arthroscopic procedures, there were a number of questions to determine clinical preferences for perioperative tourniquet and related technology use. BFR therapy is becoming more commonly incorporated in perioperative patient management.^10^^,^^23^ It is particularly valuable in prehabilitation and rehabilitation, and in the United States, it is now widely embraced by major professional sports teams for both its training and therapeutic effects.^24^ Because BFR therapy relies on tourniquet technology, it is important to understand the similarities and differences in technology and application for its safe use. Almost half of respondents already incorporate BFR therapy into their patient protocols, a third did not understand its benefit or are concerned about its risk, and others prefer to leave the treatment program to a physiotherapist. There is a substantial body of research related to the efficacy of BFR therapy, both for healthy patient populations and for perioperative patients.^10^, ^11^, ^12^^,^^23^^,^^25^, ^26^, ^27^, ^28^ Research shows that properly controlled preoperative and postoperative BFR therapy accelerates recovery after surgery and improves outcomes.^10^^,^^26^^,^^27^ Greater awareness of BFR therapy and surgery will likely result in our lessons from surgical tourniquet research being translated to BFR tourniquets and safety in implementing their use in perioperative care.

Within the preliminary forum, there was some discussion related to postoperative swelling and lymphedema. In the survey, half of participants expressed their interest in using improved technology to help reduce postoperative swelling and lymphedema and highlighted the need for further research in that area.

This study shows the potential for the AANA DocMatter Community to be a valuable mechanism for collecting data on surgical practices. The results indicate that there is a need for wider dissemination of current information and for further research on tourniquet optimization for safe use and best practices.

### Limitations

There are a number of identified limitations of this study. First, the inherent nature of circulating a survey via the DocMatter Community Forum made for a less controlled recruitment protocol and does not allow for statistical analysis of results. The DocMatter platform was intentionally selected to provide AANA Community members with the tourniquet usage prompt to allow for more casual discourse of the topic among the members. Another limitation of this study is that many of the survey questions were designed as short-form responses. Although short-form responses were enabled to allow for respondents to provide context, responses in this format do not easily allow for aggregation of results.

## Conclusions

Despite large reported variation in the indications for tourniquet use and the control of key tourniquet parameters, use is evolving through a focus on optimizing tourniquet times through selective inflation for specific aspects of arthroscopic procedures.

## Disclosures

The author (B.D.) declares that he has no known competing financial interests or personal relationships that could have appeared to influence the work reported in this paper.
